# Gastroprotective Activity of *Polygonum chinense* Aqueous Leaf Extract on Ethanol-Induced Hemorrhagic Mucosal Lesions in Rats

**DOI:** 10.1155/2012/404012

**Published:** 2012-12-26

**Authors:** Iza Farhana Ismail, Shahram Golbabapour, Pouya Hassandarvish, Maryam Hajrezaie, Nazia Abdul Majid, Farkaad A. Kadir, Fouad Al-Bayaty, Khalijah Awang, Hazrina Hazni, Mahmood Ameen Abdulla

**Affiliations:** ^1^Department of Chemistry, Faculty of Science, University of Malaya, 50603 Kuala Lumpur, Malaysia; ^2^Department of Molecular Medicine, Faculty of Medicine, University of Malaya, 50603 Kuala Lumpur, Malaysia; ^3^Institute of Biological Science, Faculty of Science, University of Malaya, 50603 Kuala Lumpur, Malaysia; ^4^Department of Anatomy, Faculty of Medicine, Cyberjaya University College of Medical Scinces, 6300 Cyberjaya, Selangor Darul Ehsan, Malaysia; ^5^Faculty of Dentistry, Universiti Teknologi Mara, 40450 Shah Alam, Malaysia

## Abstract

*Polygonum chinense* is a Malaysian ethnic plant with various healing effects. This study was to determine preventive effect of aqueous leaf extract of *P. chinense* against ethanol-induced gastric mucosal injury in rats. *Sprague Dawley* rats were divided into seven groups. The normal and ulcer control groups were orally administered with distilled water. The reference group was orally administered with 20 mg/kg omeprazole. The experimental groups received the extracts 62.5, 125, 250, and 500 mg/kg, accordingly. After sixty minutes, distilled water and absolute ethanol were given (5 mL/kg) to the normal control and the others, respectively. In addition to histology, immunohistochemical and periodic acid schiff (PAS) stains, levels of lipid peroxidation, malondialdehyde (MDA), antioxidant enzymes, and superoxide dismutase (SOD) were measured. The ulcer group exhibited severe mucosal damages. The experimental groups significantly reduced gastric lesions and MDA levels and increased SOD level. Immunohistochemistry of the experimental groups showed upregulation and downregulation of Hsp70 and Bax proteins, respectively. PAS staining in these groups exhibited intense staining as compared to the ulcer group. Acute toxicity study revealed the nontoxic nature of the extract. Our data provide first evidence that *P. chinense* extract could significantly prevent gastric ulcer.

## 1. Introduction

Gastritis is an inflammation, irritation, or erosion that occurs when the endogenous defensive mechanisms of mucosal barrier cannot properly protect the organ. Usually, exposure to exceed acid and pepsin causes insult on the gastrointestinal wall [1]. Several factors increase the incidence of peptic ulcer diseases, including *Helicobacter pylori* infection and rarely other infections such as tuberculosis, syphilis, viral infections, fungal infections, bacteria parasites, and worms. Some medications, such as NSAIDs (such as aspirin, steroids, and nonsteroidal anti-inflammatory), potassium, and iron supplements, have been reported risky for prevalence of peptic ulcer disease. In addition, medical and surgical conditions, illnesses like chronic pancreatitis, autoimmune disease, pernicious anemia, and pernicious anemia, emotional or physical distress, foods (heavy drinking alcohol), and smoking increase the chance of the commonness of the diseases among population [2]. Many products are used commonly to treat gastritis. General protocol for the treatment of gastritis is to reduce acid secretion [3]. Traditionally, plants are used for medicinal purposes in different countries. Nowadays, researchers and companies are inclined to investigate more about herbal medicines, and many plants with antiulcerogenic properties have been found by different groups [4–7].

The genus *Polygonum* belongs to Polygonaceae and comprises about 150 species. It contains various bioactive compounds and has phytopharmaceutical importance [8]. However, there was no published research about gastroprotective effect of *P. chinense* in rats. This study was undertaken to evaluate the gastroprotective potential of aqueous extract of this plant against ethanol-induced gastric mucosal hemorrhage in rats.

## 2. Materials and Methods

### 2.1. Omeprazole

In this study, omeprazole was used as a reference antiulcer drug and was obtained from the University Malaya Medical Centre (UMMC) Pharmacy. The drug was dissolved in distilled water and administered orally to the rats at a dosage of 20 mg/kg body weight (5 mL/kg) according to the recommendation of Mahmood et al. [9].

### 2.2. Plant Specimen and Preparation of Extraction

Fresh *P. chinense* leaves were obtained from Ethno Resources Sdn Bhd, Selangor, Malaysia, deposited at the Herbarium of Rimba Ilmu, Institute of Science Biology, University of Malaya, Kuala Lumpur (Voucher Specimen no. KLU 47125). The leaves were dried in shade for one week, and the dried leaves were powdered using electrical blender. The fine powder (200 g) was soaked in 1000 mL of distilled water in a conical flask and heated 1 hour on a hot plate (85°C). While its temperature was decreasing to 40° ± 5°C, it was filtered twice through cotton wool. Then, the extract was stored in a freezer. The frozen extract underwent freeze-drying process to yield dark brown powder (23.04 g, 11.52%). The extract was then dissolved in distilled water and administered orally (5 mL/kg) to rats at dosages of 62.5, 125, 250, and 500 mg/kg according to the recommendation of Mahmood et al. [10].

### 2.3. Experimental Animals and Acute Toxicity Studies

The acute toxicity study was used to determine a safe dose for *P. chinense*. Thirty-six healthy *Sprague Dawley *rats (18 males and 18 females) were obtained from the Experimental Animal House, Faculty of Medicine, University of Malaya. They were assigned equally into 3 groups: vehicle (distilled water), 2 g/kg, and 5 g/kg of the extract of *P. chinense*, respectively. The animals were fasted overnight (food but not water) prior to the dosing. Food was withheld for further 3 to 4 h after dosing. The animals were observed for 30 min and 2, 4, 24, and 48 hours after the administration for the onset of any clinical or toxicological symptoms for 2 weeks. The animals were sacrificed on the 15th day. Serum biochemical and histological (liver and kidney) parameters were determined [11]. The study was approved by the Ethics Committee for Animal Experimentation, Faculty of Medicine, University of Malaya, Malaysia with the Ethic no. PM/07/05/2011/MMA (a) (R). Throughout the experiments, all animals received human care according to the criteria outlined in the “Guide for the Care and Use of Laboratory Animals” prepared by the National Academy of Sciences and published by the National Institute of Health [11].

### 2.4. Experimental Animals for Gastric Ulcer

Healthy adult *Sprague Dawley* rats of both genders (aged between 6–8 weeks and weighed between 200–220 g) were prepared from Animal House Faculty of Medicine, University of Malaya, Kuala Lumpur, Malaysia. The animals were kept under standard laboratory conditions, caged in stainless steel cages with raised floors of wide wire mesh to prevent coprophagia, and housed at temperature of 25 ± 2°C in a 12-hour light-dark cycle. They were fed with standard laboratory pellet and water *ad libitum*. The animals were fasted for 24 hours before treatment to ensure their stomachs are empty. During the fasting period, the rats were allowed to have free access of water. Their access to water was inhibited for 2 hours before the experiment triggered. This protocol was based on the guidelines of Animal Care and Use Committee from Laboratory Animal Science Centre, Faculty of Medicine, University of Malaya, Kuala Lumpur, Malaysia.

#### 2.4.1. Induction of Gastric Ulcer

The following pretreatment was according to the recommendations of Mahmood et al. [9]. The fasted rats were divided randomly into 7 groups of 6 rats. The groups were numbered 1–7 and the pretreatment began accordingly. Group 1 served as the normal control and received distilled water orally. Group 2 labeled “ulcer control group” received distilled water orally as a pre-treatment. Group 3, the reference group, received 20 mg/kg omeprazole orally. The experimental groups (group 4–7) received aqueous leaf extract of *P. Chinense, *at dosages of 62.5, 125, 250, and 500 mg/kg, respectively. An hour after the pre-treatment, distilled water (5 mL/kg) was orally administered to the group 1, and absolute ethanol (5 mL/kg) was orally given to groups 2–7. After 1 hour, all animals were euthanized by an overdose of xylazine and ketamine anesthesia followed by cervical dislocation technique to assure their euthanasia. Their stomachs were immediately excised and were kept in containers of normal saline [6].

#### 2.4.2. Determination of Gastric Wall Mucus

The gastric wall mucus was evaluated according to the modified procedure of Corne et al. [12].

#### 2.4.3. Gross Evaluation of Gastric Lesions

Ulcers of the gastric mucosa appear as elongated bands of hemorrhagic lesions parallel to the long axis of the stomach. Gastric mucosa of each rat was examined for estimate damage. The length and width of the ulcer (mm) were measured with a planimeter (10 × 10 mm^2^ = ulcer area) under a dissecting microscope (1.8x). The ulcerated area was calculated through totaling the number of small squares (2 × 2 mm^2^) covering an ulcer band. The sum of the lesions areas for each stomach was applied in the calculation of the ulcer area (UA), where “the sum of small squares × 4 × 1.8 = UA (mm^2^)” according to the recommendation of Mahmood et al. [10]. The inhibition percentage (*I*%) was calculated by the following formula:

(1) $$\begin{array}{c} \left(I\%\right)=\left[\frac{\left({\text{UA}}_{\text{control}}-{\text{UA}}_{\text{treated}}\right)}{{\text{UA}}_{\text{control}}}\right]\times 100\%. \end{array}$$

#### 2.4.4. Preparation of Homogenate

The gastric tissue samples were washed thoroughly with ice-cold saline. Homogenates (10% (w/v)) were then prepared with ice-cold 50 mM phosphate buffer (pH 7.4) containing mammalian protease inhibitor cocktail. The homogenates were centrifuged at 10,000 ×g for 30 min (4°C). The supernatant was used for further experiments.

#### 2.4.5. Measurement of SOD Activity

SOD activity was measured according to the protocol of Sun et al. [13].

#### 2.4.6. Measurement of MDA

Tissue MDA (mMol/L) was determined based on the method developed by Draper and Hadley [14].

#### 2.4.7. Measurement of Protein Concentration

Biuret reaction was the main protocol to measure the protein concentrations, as described by Gornall et al. [15].

### 2.5. Histological Studies of the Gastric Mucosa 

#### 2.5.1. Preparation of Tissue Sections

Specimens of the gastric walls were fixed in 10% buffered formalin for 18 hours at room temperature and processed by a tissue-processing machine (Leica, Germany). Sections of the stomach were adopted at a thickness of 5 *μ*m.

#### 2.5.2. Hematoxylin and Eosin

Stomach sections were stained with Hematoxylin and Eosin for histological evaluation [16].

#### 2.5.3. Study of Mucosal Glycoproteins

The glandular portion of stomach was stained with periodic acid schiff (PAS) for each rat in each group [17].

### 2.6. Immunohistochemical Staining

Tissue section slides were heated at 60°C for approximately 25 min in a hot air oven (Venticell, MMM, Einrichtungen, Germany). The tissue sections were deparaffinized in xylene and rehydrated with graded alcohol. Antigen retrieval process was performed in a 10 mM sodium citrate buffer. Immunohistochemical staining was conducted according to manufacturer's protocol (Dakocytomation, USA). Briefly, endogenous peroxidase was blocked by peroxidase block (0.03% hydrogen peroxide containing sodium azide) for 5 min. Tissue sections were washed gently with wash buffer and then incubated with Hsp70 (1 : 500) and Bax (1 : 200) biotinylated primary antibodies for 15 min. The sections were rinsed gently with wash buffer and placed in buffer bath. The slides were then placed in a humidified chamber and sufficient amount of streptavidin—HRP (streptavidin conjugated to horseradish peroxidase in PBS containing an antimicrobial agent) was added and incubated for 15 min. Then, tissue sections were rinsed gently in wash buffer and placed in buffer bath. Diaminobenzidine-substrate-chromagen was added to the tissue sections and incubated further for 5 min following washing and counterstaining with hematoxylin for 5 seconds. The sections were then dipped in weak ammonia (0.037 Mol/L) 10 times and then rinsed with distilled water and cover slipped. Positive findings of the immunohistochemical staining should be seen as brown stains under light microscope.

### 2.7. Statistical Analysis

All values were reported as mean ± S.E.M. The statistical significance of differences between groups was assessed with one-way ANOVA (post hoc analysis). A value of *P* < 0.05 was considered significant.

## 3. Results

### 3.1. Acute Toxicity Study

To determine the acute toxicity of the *P. chinense *extract, the animals were treated with the extract at a dose of 2 g/kg or 5 g/kg. Their health conditions were screened for a period of 14 days. All of the animals were healthy and did not manifest any sign of toxicity at these doses. Serum biochemical and histologic indicators of liver and kidney did not show any abnormalities.

### 3.2. Gross Evaluation of Gastric Lesions

**Figure 1:**
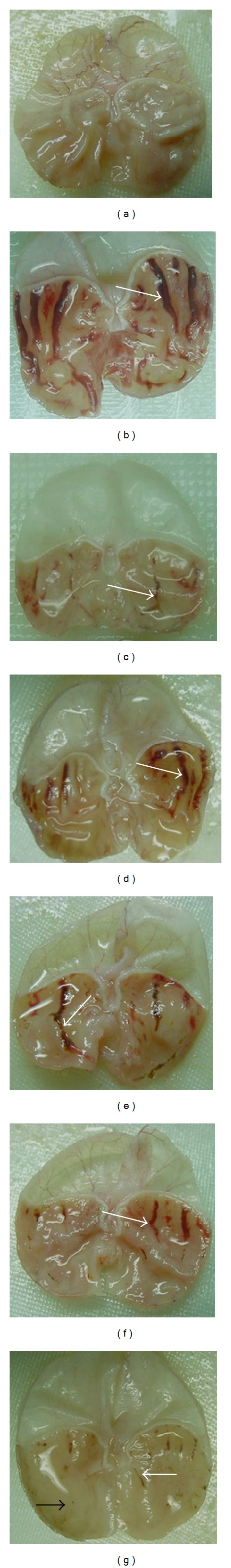
Macroscopic appearance of gastric mucosal lesions of the rats in different groups. (a) Rats in the normal control group showed intact gastric mucosa. (b) The ulcer control group (pretreated with 5 mL/kg absolute alcohol) showed severe injuries to the gastric mucosa (white arrow). Absolute ethanol imposed extensive hemorrhagic necrosis to gastric mucosa. (c) The reference group (omeprazole 20 mg/kg) showed milder injuries to the gastric mucosa (white arrow) compared with the ulcer control rats. (d) Rat pre-treated with *P. chinense* (62.50 mg/kg) showed moderate injuries on the gastric mucosa (white arrow). (e) Pre-treated with 125 mg/kg of *P. chinense* extract suppressed lesions to a mild-moderate condition (white arrow). (f) Mild injuries of the gastric mucosa were found in those rats pre-treated with 250 mg/kg of *P. chinense* extract. (g) Pre-treatment with 500 mg/kg of the extract protected injuries at a mild condition and flattened the gastric mucosa observed (black arrow).

The anti-ulcer activity of *P. chinense* extract in ethanol-induced hemorrhagic mucosal lesions model is shown in Figure 1 and Table 1. The lesions were long, hemorrhagic, and confined to the glandular portions. Results showed that those rats pre-treated with the extract significantly reduced areas of gastric ulcer formation when compared with the ulcer control group (Figure 1). Absolute ethanol caused extensive and visible hemorrhagic lesions to gastric mucosa. The *P. chinense* extract significantly suppressed the formation of the ulcers, especially at its highest dosage, and astoundingly flattened gastric mucosal folds at the dosage of 500 mg/kg of the extract (Figure 1). The extract significantly reduced the ulcer size and severity in a dose-dependent manner. The protection properties of the extract at its highest dosage (500 mg/kg) appeared similar to the reference group (Figure 1).

### 3.3. Effect of **P. chinense ** on Ethanol-Induced Changes in Gastric Wall Mucus

In the ulcer control group, in comparison to the normal control group, ethanol significantly decreased the Alcian-blue-binding capacity of gastric wall mucus. In the experimental groups, pre-treatment with the *P. chinense *extract significantly enhanced the Alcian-blue-binding capacity of the gastric mucosa (Table 1). 

### 3.4. Effect of *P. chinense* on Measurements of SOD Activity

Ethanol was able to reduce SOD activity as shown in the ulcer control group, compared to normal control group. In the experimental groups, the extract of *P. chinense* caused a significant increase in the enzyme activity of SOD (Table 1). 

### 3.5. Effect of *P. chinense* Extract on Tissue MDA

Ethanol increased the MDA activity significantly when compared with the normal group. However, the extract of *P. chinense *significantly decreased MDA activity, similar to the reference group (Table 1).

### 3.6. Effect of *P. chinense* Extract on Protein Concentration

In the ulcer control group, ethanol significantly decreased the protein concentration of the gastric mucosal homogenate when compared with the normal control group. However, increase in the protein concentration was evident in the experimental groups as it was in those rats receiving omeprazole (Table 1).

### 3.7. Histological Evaluation of Gastric Lesions

**Figure 2:**
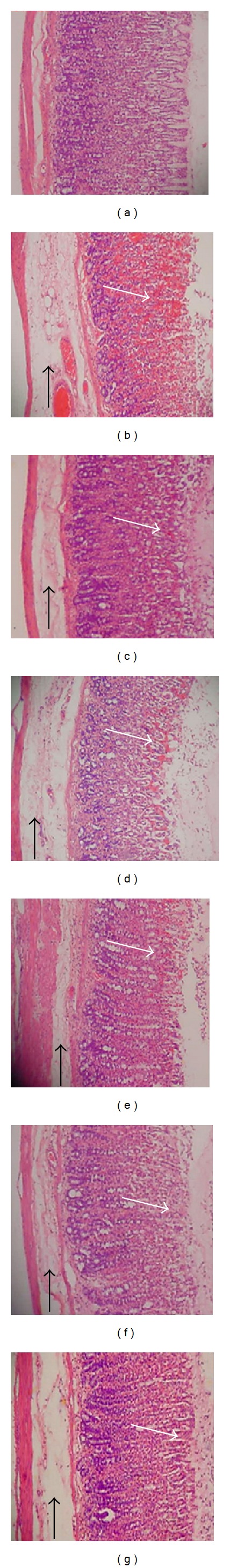
Histology evaluation of gastric mucosal lesions of the rats in different groups (H&E staining 10x). (a) Rats in the normal control group showed intact gastric mucosa. (b) The ulcer control group (pre-treated with 5 mL/kg absolute alcohol) showed severe disruption on the epithelium. The necrotic lesions penetrated deeply into mucosa (white arrow). Also, extensive edema and leucocyte infiltration of submucosal layer were seen (black arrow). (c) The reference group (omeprazole 20 mg/kg) showed a mild disruption of the epithelium with edema and leucocyte infiltration of submucosal layer. (d) Rat pre-treated with *P. chinense* (62.50 mg/kg) showed a moderate disruption of epithelium with edema and leucocytes infiltration of submucosal layer. (e) Pre-treated with 125 mg/kg of *P. chinense* extract suppressed disruption of surface epithelium with edema and leucocyte infiltration of submucosal layer to a mild-moderate condition. (f) Mild disruption of the epithelium was found in those rats pre-treated with 250 mg/kg of *P. chinense* extract. (g) Pre-treatment with 500 mg/kg of the extract protected the epithelium from disruption at a mild condition along with a mild edema and leucocytes infiltration of the submucosal layer.

Histological observation of gastric lesions in the ulcer control group showed comparatively extensive damage of the gastric mucosa, and necrotic lesions penetrated deeply into the mucosa. Also, extensive oedema and leucocytes infiltrations of the submucosal layer appeared microscopically (Figure 2). Rats receiving the extract of *P. chinense* had better protection for the gastric mucosa from reduction or absence of ulcer area, submucosal oedema, and leucocytes infiltration (Figure 2). The extract showed its potential to exert the gastroprotective effect in a dose-dependent manner.

### 3.8. Periodic Acid Schiff (PAS) of Mucosal Glycoproteins

**Figure 3:**
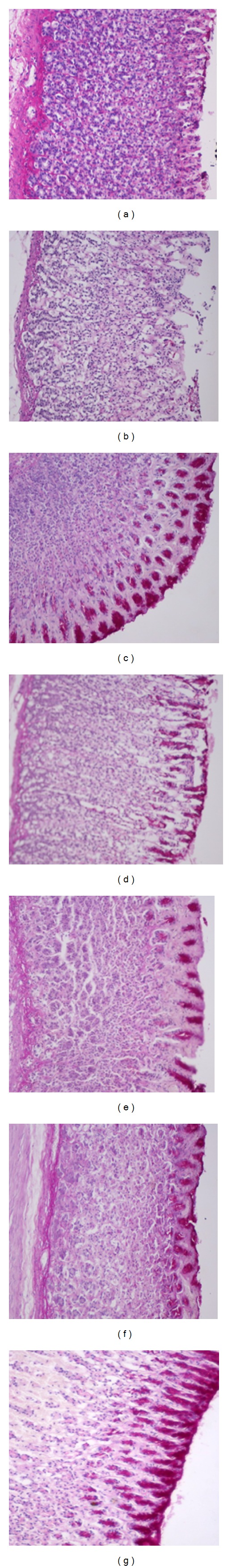
PAS staining evaluation of gastric mucosal lesions of the rats in different groups (PAS staining 20x): (a) the normal control group; (b) the ulcer control group; (c) the reference group (omeprazole 20 mg/kg); (d). rat pre-treated with *P. chinense* (62.50 mg/kg); (e) rat pre-treated with *P. chinense* (125 mg/kg); (f) rat pre-treated with *P. chinense* (250 mg/kg); (g) rat pre-treated with *P. chinense* (500 mg/kg).

The extract of *P. chinense* increased the PAS staining of gastric mucosa, as evident in Figure 3 (the magenta color), in comparison to the ulcer control group, indicating the increase in glycoprotein content of gastric mucosa. In the other word, the *P. chinense* extract reserved the decrease in PAS staining induced by absolute ethanol (Figure 3).

### 3.9. Immunohistochemistry

**Figure 4:**
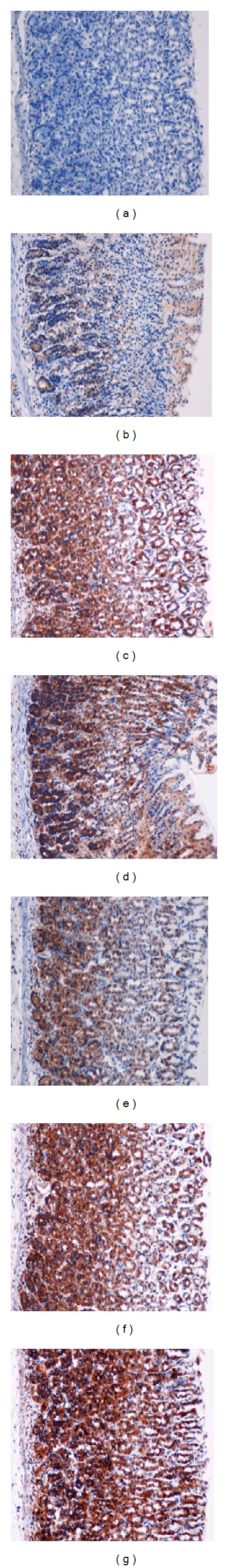
Immunohistochemical evaluation of expression of Hsp70 protein appearance of gastric mucosal lesions of the rats in different groups (20x): (a) the normal control group; (b) the ulcer control group; (c) the reference group (omeprazole 20 mg/kg); (d). rat pre-treated with *P. chinense* (62.50 mg/kg); (e) rat pre-treated with *P. chinense* (125 mg/kg); (f) rat pre-treated with *P. chinense* (250 mg/kg); (g) rat pre-treated with *P. chinense* (500 mg/kg).

**Figure 5:**
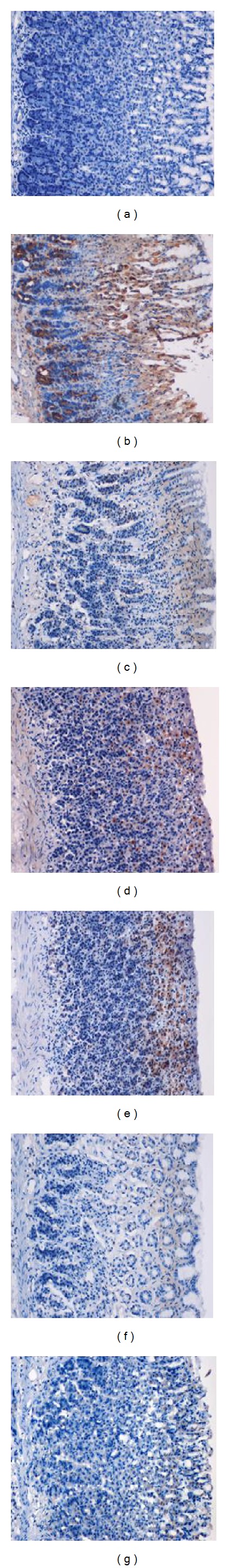
Immunohistochemical evaluation of expression of Bax protein appearance of gastric mucosal lesions of the rats in different groups (20x): (a) the normal control group; (b) the ulcer control group; (c) the reference group (omeprazole 20 mg/kg); (d) rat pre-treated with *P. chinense* (62.50 mg/kg); (e) rat pre-treated with *P. chinense* (125 mg/kg); (f) rat pre-treated with *P. chinense* (250 mg/kg); (g) rat pre-treated with *P. chinense* (500 mg/kg).

Immunohistochemistry results showed that the pre-treatment with the *P. chinense *extract caused upregulation of Hsp70 protein. Similarly, omeprazole enhanced the expression of Hsp70. The expression of Hsp70 protein in the ulcer control group underwent downregulation when compared to that of experimental groups (Figure 4). Bax protein, on the other hand, in the animals pre-treated with the *P. chinense *extract (as well as those rats in the reference group) was downregulated. In contrast, the expression of Bax protein in the ulcer control group was upregulated (Figure 5). Bax positive cells are characterized by the brown staining in the cytoplasm of epithelial cells in the gastric glands.

## 4. Discussions

The acute toxicity test did not show any signs of toxicity or mortality in the applied dosages. Oral administration of absolute ethanol was noxious to the stomach. It caused topical disruption of gastric mucosa barrier and provoked remarkable vascular changes within a few minutes [16]. Absolute ethanol also produced linear hemorrhagic lesions, extensive submucosal edema, mucosal friability, inflammatory cells infiltration, and epithelial cell loss into the gastric lumen, typical characteristics of alcoholic injuries [18]. Mucus secretion was assumed a crucial defensive factor to protect the gastric mucosa from gastric lesions [19]. 

Administration of ethanol, in ulcer control group, reduced protein concentration, but oral pre-treatment with the plant extract maintained protein concentration in the gastric homogenates. Ethanol could damage epithelial cells, leading to reduction of protein concentration [20]. Mucus membrane, the first defending layer of the stomach tissue, could be eroded by ethanol. Gastric mucosa prevented direct contact to the digestive enzymes [20]. The extract of *P. chinense* could enhance generation of epithelial cells which in turn significantly increased the protein concentration of the gastric homogenates.

Superoxide and hydroxyl radicals are important mediators of oxidative stress that play vital role in some clinical disorders. Any compounds (natural or synthetic) with antioxidant activities might contribute towards the total/partial alleviation of such damage. Therefore, eliminating superoxide and hydroxyl radical could contribute to defend a living body against disease [21]. SOD converts superoxide to hydrogen peroxide, and subsequently catalase converts hydrogen peroxide to water. The gastric mucosal homogenate in the ulcer control group reduced the activity of SOD. This might be resulted from their utilization for the decomposition of superoxide anion, generated by lipid peroxidation. Lowered activity of this enzyme may end up to a number of deleterious results. The pre-treatment with the *P. chinense* extract increased the activity of SOD. In the stomach homogenates, the *P. chinense* extract significantly decreased the concentration of malondialdehyde, an indicator for lipid peroxidation. Lipid peroxidation is a recognized example of oxidative damage that affects cell membranes. It is caused by an imbalance between oxidative damage and antioxidant defense systems. The reduction in lipid peroxidation by the extract pointed to its antioxidant activity. Moreover, *P. chinense* extract might protect/prevent significant changes in biochemical parameters and morphologic changes of gastric mucosa when absolute ethanol was administrated. MDA level was significantly increased in the ulcer group along with reduction in SOD antioxidant enzyme activity. 

Histological evaluation of the gastric tissue showed that the characteristics of ethanol-induced lesions consist of hemorrhage, edema, inflammatory infiltrate, and loss of epithelial cells as previously reported by other studies [22, 23]. Result of the present study proved the gastroprotective property of the *P. chinense* extract in the company of inhibition of leucocytes infiltration to preserve the gastric wall. Absolute alcohol could extensively damage the gastric mucosa and could increase neutrophil infiltration into the gastric mucosa. Oxygen free radicals, derived from infiltrated neutrophils in ulcerated gastric tissues, have inhibitory effect on gastric ulcers healing in rats. Neutrophils also mediate lipid peroxidation through the production of superoxide anions [24]. Neutrophils as a main source of inflammatory mediators can release potent reactive oxygen species (such as superoxide, hydrogen peroxide, and myeloperoxidase derived oxidants). These reactive oxygen species are highly cytotoxic and can induce tissue damage [25].

In this study, the flattening of the mucosal folds appeared as an utmost gastroprotective effect of the *P. chinense* leaf extract which might be due to the reduced motility of the stomach. Changes in the gastric motility might influence the incidence of gastric lesions [4]. Relaxation of circular muscles could protect the gastric mucosa through the flattening of the mucosal folds which might increase the mucosal area exposed to necrotizing agents and would reduce the volume of the gastric irritants on rugal crest [6]. Ethanol triggered a remarkable contraction of the circular muscles and led to mucosal compression at the crests of mucosal folds, leading to necrosis and ulceration [10]. 

The PAS staining method exhibited characteristic carmine staining of stomach regions that secreted mucopolysaccharides. Our study clarified that the *P. chinense* extract enhanced intense secretion of the mucus from gastric glands. The mucus production is one of the main mechanisms of local gastric mucosal defense [24]. Among various factors enhancing ulcer prevention in stomach, mucus and bicarbonate secretion might be extremely important in the ulcer preventing process as they produced a mucus/bicarbonate layer protecting newly formed cells from irritations (such as acid and peptic injury) [26].

Apoptosis, programmed death of cells through DNA fragmentation, cell shrinkage, and dilation of endoplasmic reticulum are normally followed by cell degeneration and the formation of membrane vesicles, called apoptosis bodies. Ethanol by itself is able to induce apoptosis in the gastric epithelium in the late phase of its imposition. The upregulation of proapoptotic factor, Bax protein, and downregulation of antiapoptotic Bcl-2 are two main indicators for the apoptosis. Hsp70, on the other hand, is the most conserved and abundantly produced protein in response to different forms of stress [27], such as heat, toxic agents, infection, and proliferation [28]. Interaction between these proteins is important in maintaining the cellular homeostatic state [29]. Hsp70 proteins defend cells from oxidative stress or heat shock. Ethanol-generated reactive oxygen species normally act to inhibit the expression of Hsp70 and increase the expression of Bax. Hsp70 prevents these partially denatured proteins from aggregating and allows them to refold. The overexpression of Hsp70 noticed in this study could suggest that the *P. chinense* protected the gastric tissues through the up-regulation of Hsp70. 

The Hsp70 family functions as a molecular chaperone and reduces stress-induced denaturation and aggregation of intracellular proteins. In addition to its chaperoning activities, Hsp70 has been suggested to exert its gastroprotective action by protecting mitochondria and by interfering with the stress-induced apoptotic program [30]. Animals pre-treated with *P. chinense *extract showed down expression of Bax protein. Bax protein was found to be up-regulated in ulcer control group. Bax is one of the Bcl-2 family and known to be a key protein relating to apoptosis through mitochondrial injury [31]. The susceptibility of a cell to apoptosis depends on the balance between apoptosis-promoting and -suppressing factors [32]. Apoptotic cell death plays significant roles in the loss of gastric mucosal integrity caused by various aggressive factors [33]. Significant induction of gastroprotective Hsp70 was found in the *P. chinense*-administrated rats, suggesting that the restoration of the proteins might contribute to prevention of ethanol-induced gastric hemorrhagic mucosal lesions. It is, therefore, suggested that this plant has a protective effect against ethanol-induced gastric damages by induction of Hsp70. 

## 5. Conclusions

Our study reveals that *P. chinense* leaf extract could significantly protect gastric mucosa against ethanol-induced gastric mucosal injury. Such protection was shown as ascertain by gross appearance, histology, PAS, and immunohistochemistry staining gastric tissue homogenate, which is related partly to a preservation of gastric mucus secretion and to the antioxidant activity. 

## Figures and Tables

**Table 1:** The ulcer area, percentage of inhibition, gastric wall mucus (mg Alcian blue/g tissue), MDA (*μ*mol/g protein), SOD (U/g protein), and protein concentration (mg/mL tissue) of the groups.

Groups	Ulcer area	Inhibition %	GWM	MDA	Protein	SOD
1	—	—	245.13 ± 2.2*	70 ± 2.93*	10 ± 0.16*	236.13 ± 4.29*
2	921.90 ± 16.68	—	125.10 ± 2.3	228 ± 4.53	5.17 ± 0.12	121.15 ± 2.50
3	159.30 ± 5.04*	82.72	320.11 ± 2.4*	95.08 ± 3.3*	8.45 ± 0.16*	423.00 ± 5.83*
4	455 ± 12.16*	50.64	282.00 ± 1.8*	110 ± 4.23*	7.22 ± 0.06*	285.00 ± 4.83*
5	315.23 ± 9.17*	65.81	303.1 ± 2.5*	108 ± 3.65*	7.86 ± 0.04*	350.10 ± 4.29*
6	235.61 ± 7.39*	74.44	315.08 ± 2*	102.1 ± 3.7*	9.40 ± 0.12*	402.42 ± 5.95*
7	114.00 ± 5.41*	87.63	325.11 ± 4.4*	92.15 ± 3.2*	9.70 ± 0.14*	415.13 ± 6.90*

Values are assumed as mean ± S.E.M. The statistical analysis was assessed with one-way ANOVA (post hoc analysis) with *P* < 0.05. *Significant differences when compared to group 2. (1) The normal control group. (2) the ulcer control group. (3) the reference control. (4) 62.50 mg/kg of the plant extract; (5) 125 mg/kg of the plant extract. (6) 250 mg/kg of the plant extract. (7) 500 mg/kg of the plant extract.
