# Corrigendum to “Gastroprotective Activity of* Polygonum chinense* Aqueous Leaf Extract on Ethanol-Induced Hemorrhagic Mucosal Lesions in Rats”

**DOI:** 10.1155/2018/8961462

**Published:** 2018-12-02

**Authors:** Iza Farhana Ismail, Shahram Golbabapour, Pouya Hassandarvish, Maryam Hajrezaie, Nazia Abdul Majid, Farkaad A. Kadir, Fouad Al-Bayaty, Khalijah Awang, Hazrina Hazni, Mahmood Ameen Abdulla

**Affiliations:** ^1^Department of Chemistry, Faculty of Science, University of Malaya, 50603 Kuala Lumpur, Malaysia; ^2^Department of Molecular Medicine, Faculty of Medicine, University of Malaya, 50603 Kuala Lumpur, Malaysia; ^3^Institute of Biological Science, Faculty of Science, University of Malaya, 50603 Kuala Lumpur, Malaysia; ^4^Department of Anatomy, Faculty of Medicine, Cyberjaya University College of Medical Scinces, 6300 Cyberjaya, Selangor Darul Ehsan, Malaysia; ^5^Faculty of Dentistry, Universiti Teknologi Mara, 40450 Shah Alam, Malaysia

In the article titled “Gastroprotective Activity of* Polygonum chinense* Aqueous Leaf Extract on Ethanol-Induced Hemorrhagic Mucosal Lesions in Rats” [1], it was found that Figure 2(e) is the same as Figure 3(g) in another article by the same authors, Golbabapour et al. [2], when rotated and flipped.

An institutional investigation by the University of Malaya found there was no system to index and file data and images to avoid mislabeling and mishandling, which led to errors and duplication of research data. The authors did not thoroughly check the manuscript before submission.

The authors explained that the two articles had the same negative control group, as suggested by the ethics committee to reduce the number of laboratory animals used. The image in Figure 3(g) of [2] is correct. Therefore, Figure 2(e) should be corrected with one of the original captures of the same histological tissue. The rotation of the images was applied to frame the images vertically.

The corrected Figure 2, with Figure 2(e) replaced, is as shown below:

## Figures and Tables

**Figure 2:**
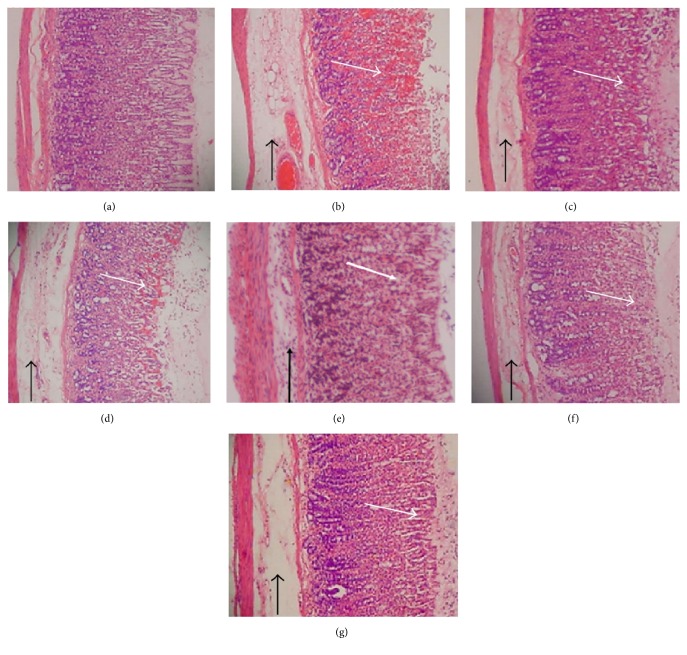
Histology evaluation of gastric mucosal lesions of the rats in different groups (H&E staining 10x). (a) Rats in the normal control group showed intact gastric mucosa. (b) The ulcer control group (pretreated with 5 mL/kg absolute alcohol) showed severe disruption on the epithelium. The necrotic lesions penetrated deeply into mucosa (white arrow). Also, extensive edema and leucocyte infiltration of submucosal layer were seen (black arrow). (c) The reference group (omeprazole 20 mg/kg) showed a mild disruption of the epithelium with edema and leucocyte infiltration of submucosal layer. (d) Rat pretreated with P. chinense (62.50 mg/kg) showed a moderate disruption of epithelium with edema and leucocytes infiltration of submucosal layer. (e) Pretreated with 125 mg/kg of P. chinense extract suppressed disruption of surface epithelium with edema and leucocyte infiltration of submucosal layer to a mild-moderate condition. (f) Mild disruption of the epithelium was found in those rats pretreated with 250 mg/kg of P. chinense extract. (g) Pretreatment with 500 mg/kg of the extract protected the epithelium from disruption at a mild condition along with a mild edema and leucocytes infiltration of the submucosal layer.
